# Endocannabinoid Tone and Oxylipins in Rheumatoid Arthritis and Osteoarthritis—A Novel Target for the Treatment of Pain and Inflammation?

**DOI:** 10.3390/ijms26125707

**Published:** 2025-06-14

**Authors:** Jost Klawitter, Andrew D. Clauw, Jennifer A. Seifert, Jelena Klawitter, Bridget Tompson, Cristina Sempio, Susan L. Ingram, Uwe Christians, Larry W. Moreland

**Affiliations:** 1 Department of Anesthesiology, School of Medicine, University of Colorado Anschutz Medical Campus, Aurora, CO 80045, USA; jelena.klawitter@cuanschutz.edu (J.K.); bridget.tompson@cuanschutz.edu (B.T.); cristina.sempio@cuanschutz.edu (C.S.); susan.ingram@cuanschutz.edu (S.L.I.);; 2 Department of Psychiatry, School of Medicine, University of Colorado Anschutz Medical Campus, Aurora, CO 80045, USA; 3 Division of Rheumatology and Clinical Immunology, School of Medicine, University of Colorado Anschutz Medical Campus, Aurora, CO 80045, USA; andrew.clauw@cuanschutz.edu (A.D.C.); jennifer.seifert@cuanschutz.edu (J.A.S.); larry.moreland@cuanschutz.edu (L.W.M.); 4 Division of Renal Diseases and Hypertension, School of Medicine, University of Colorado Anschutz Medical Campus, Aurora, CO 80045, USA

**Keywords:** endocannabinoid system, biomarkers, osteoarthritis, rheumatoid arthritis, pain, inflammation

## Abstract

Inflammation is a complicated physiological process that contributes to a variety of disorders including osteoarthritis (OA) and rheumatoid arthritis (RA). Endocannabinoids and the endocannabinoid system (ECS) play a pivotal role in the physiological response to pain and inflammation. A clinical study to investigate the role of the endocannabinoid system and related lipids in pain and inflammation in OA and RA was performed. In total, 80 subjects, namely, 25 patients with RA, 18 with OA, and 37 healthy participants, were included. Sixteen endocannabinoids and congeners, as well as 129 oxylipins, were quantified in plasma using specific, quantitative LC-MS/MS assays. The endocannabinoid analysis revealed significantly lower levels of 2-arachidonoylglycerol (2-AG) in RA and OA patients compared to healthy participants. In contrast, the EC levels of the ethanolamide group (anandamide, docosahexaenoyl-EA, palmitoleoyl-EA, and other ethanolamides) were higher in the RA study cohort and to a lesser extent also in the OA cohort. This analysis of oxylipins revealed lower levels of the pro-resolving lipid 9-oxo-octadecadienoic acid (9-oxoODE) and the ω-3 fatty acids EPA (eicosapentaenoic acid) and DHA (docosahexaenoic acid) in RA compared to all other study cohorts. 2-AG is a key regulator of nociception and inflammation, and its relatively low levels might be a mechanistic contributor to residual pain and inflammation in RA and OA. Several changes in pro- and anti-inflammatory lipid mediators were detected, including lower levels of EPA and DHA in RA, which might reveal the potential for nutritional supplementation with these anti-inflammatory fatty acids.

## 1. Introduction

Arthritis, derived from the Greek term “disease of the joints”, is defined as an acute or chronic joint inflammation that often co-exists with pain and structural damage [1,2]. There are several types of arthritis, the most common of which are osteoarthritis (OA), rheumatoid arthritis (RA), juvenile idiopathic arthritis, gout, and fibromyalgia [3]. The United States Centers for Disease Control and Prevention (CDC) estimate that 1 in 5 (or 53.2 million) adults in the United States have some form of arthritis [4]. The high prevalence of arthritis manifests in enormous societal and personal costs. Of the different forms of arthritis, osteoarthritis is most prevalent, affecting 32.5 million adults in the United States [5,6]. This number is bound to increase with the expected increase in mean population age [3,7,8]. OA is a whole-joint disease, involving all articular structures, including cartilage and meniscal degeneration, subchondral bone remodeling, and inflammation of both the infrapatellar fat pad and the synovial membrane [9,10]. Osteoarthritis represents a failed repair of joint damage resulting from stresses initiated by any joint or periarticular tissue abnormality [11]. The major contributory factors to OA include advanced age [12], female sex [12], joint trauma [13], occupational factors (e.g., knee bending, heavy lifting, and squatting) [13,14,15], obesity [13,16], and genetic factors [11,17,18]. In comparison, RA is a systemic autoimmune inflammatory disorder. The lifetime risk of developing RA in adults is 3.6% for women and 1.7% for men in the United States [19]. RA is clearly associated with high levels of inflammation, whereas inflammation plays a less pronounced role in OA [20].

For OA, primary treatments provided to delay surgery and manage pain in hip OA are intra-articular corticosteroids and exercise [11,21]. Pain treatment therapeutic options for patients with OA are limited and focus mostly on nonsteroidal anti-inflammatory drugs (topical and oral) [11]. Opioids are still often prescribed for OA pain, despite recommendations to limit use due to minimal benefits and associated harms [22]. Opioids not only possess analgesic and antinociceptive properties, but also have a strong rewarding effect, which may lead to addiction. The chronic use of opioids can also produce opioid-induced hyperalgesia [23]. Rheumatoid arthritis therapy focuses on targeting the immune system’s signaling molecules, such as tumor necrosis factor (TNF) and interleukin 6 (IL-6), which are involved in the progression of the disease [24]. Although TNF-targeting drugs have been found to be effective in most patients, they fail to achieve the desired clinical outcome or sustain the positive response in about 30–40% of patients, due to their adverse effects [25]. These targeted treatment therapeutic agents are called biological disease-modifying antirheumatic drugs (bDMARDS) and include abatacept, tocilizumab, and rituximab. But there are disadvantages for these molecular targeted therapies including high costs of production and parenteral administration [24,25].

Over the past 50 years, the endocannabinoid system has emerged as an important endogenous system involved in a wide range of physiological and pathological processes [26]. The ECS is composed of cannabinoid receptors (CBRs), their endogenous lipid-based ligands (endocannabinoids), and their cognate synthetic and degradative enzymes [26]. Cannabinoid receptors CB_1_ and CB_2_ are expressed in the central nervous system and peripheral tissues [27,28]. It has been shown that CB_1_ and CB_2_ receptors are expressed in human chondrocyte [29], human synovial tissue [30], and human fibroblast-like synoviocyte cultures [31,32]. Other proposed cannabinoid/endocannabinoid receptors include the G-protein coupled receptor 55 (GPR55) [33], transient receptor potential vanilloid 1 (TRPV1) ion channel [34], and peroxisome proliferator-activated receptor (PPAR) α and γ [35]. In recent years, studies have found that the ECS plays a pivotal role in physiological processes including pain, inflammation, immune function, neurological function, and body homeostasis. While there is increasing interest in the therapeutic potential of the pharmacological targeting of cannabinoid receptors [36], relatively little is known about the role of endocannabinoids and CBRs in RA and OA in patients undergoing current treatment protocols. In this context, it is reasonable to expect that a better understanding of the role of the endocannabinoid system (ECS) in OA and RA as a potential novel treatment target, alone or in addition to common therapies, will be critically important. We utilized a targeted high-performance liquid chromatography–tandem mass spectrometry (LC-MS/MS)-based lipidomics approach in combination with a bead-based multiplex cytokine immunoassay to identify potential relationships among endocannabinoids, related lipid mediators, and cytokines associated with OA and RA in plasma collected from patients undergoing treatment.

## 2. Results

### 2.1. Study Results

#### 2.1.1. Demographics and Drug Use

Table 1 summarizes the study population characteristics. Race and sex were approximately equally distributed across the three study cohorts (Table 1). The median age of all study participants was 56 years. The mean age was slightly higher in patients with OA (62 ± 7 years) than with RA (57 ± 8 years) as compared to the healthy controls (52 ± 12 years), but not statistically significant. The present study did not include de novo diagnosed patients with RA and OA, and there were differences in medication use amongst the study cohorts. The most pronounced difference was that all patients with RA used disease-modifying antirheumatic drugs (DMARDs), and several of said patients used more than one DMARD (Table 2). The DMARDs used in the RA cohort included methotrexate (n = 11), adalimumab (n = 6), etanercept (4), sulfasalazine (n = 3), hydroxychloroquine (n = 2), abatacept (n = 2), leflunomide (n = 2), rituximab (n = 1), tofacitinib (n = 1), upadacitinib (n = 1), and sarilumab (n = 1). Sex, age, and race were included as factors in the statistical models, but there were no effects of these variables on the plasma levels of endocannabinoids, lipid mediators, and cytokines. Therefore, it was not further differentiated between sex, age, and race within the study cohorts. Medication was included as a factor in the statistical models. All participants of the RA cohort received disease-modifying antirheumatic drugs (DMARDs) as part of their standard care. Most patients with OA used NSAIDs in addition to several other medications, whereas only one subject of the healthy control group used NSAIDs. Thus, the effects observed for the RA group are associated with DMARD use and those for the OA group with the use of NSAIDs and other medications too.

#### 2.1.2. Cytokine Analysis

**Figure 1:**
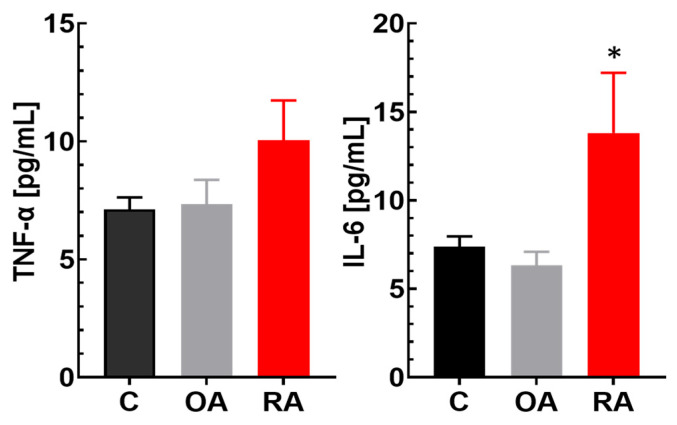
Key inflammatory cytokines for the development and severity of RA. Values for osteoarthritis (OA) and rheumatoid arthritis (RA) compared to healthy controls (C). For abbreviations, please see the text. Data are shown as mean ± SEM. * FDR < 0.05.

Of the 46 cytokines included in the human Cytokine XL Fixed Panel MAGPIX assay, the majority were excluded from the analysis since the levels were below the detection limits of the assay: MIP-1β, CD40 Ligand, GROα, TGF-α, TRAIL, FGF basic, G-CSF, GM-CSF, Granzyme B, IFN-α2, IFN-β, IL-1α, IL-1β, IL-2, IL-3, IL-4, IL-5, IL-7, v8, IL-9, v10, IL-12 P70, v15, IL-17A, IL-17B, and IL-33. The results of the analysis of cytokines that were in the quantifiable range of the assay are listed in Supplementary Table S1. Interleukin IL-6 and TNFα are included in Supplementary Table S1, because these are considered key pro-inflammatory cytokine biomarkers known to be increased in de novo patients with RA [37,38], although their values were below the concentration of the lowest calibrator for the majority of all samples, but were above the limit of detection. As mentioned above, the patients in our study were not de novo patients with OA or RA, and all participants in the RA group received DMARDs as part of the treatment. Therefore, it was not surprising to observe unquantifiable/low levels for many pro-inflammatory cytokines. Others, such as EGF, GROβ, PDGF-AA, PDGF-AB/BB, and RANTES, were statistically significantly lower in patients with RA than in healthy participants and also mostly lower in patients with OA (Supplementary Table S1). The cytokines that were still elevated and not as much affected by the therapy in RA as compared to healthy participants were Flt-3 Ligand, IL-1ra, and MCP-1 (Supplementary Table S1). Evidence from previous studies suggests that TNF-α and IL-6 play a significant role in the occurrence and development of RA due to their pro-inflammatory effects [39,40,41]. TNF-α and IL-6 plasma levels for healthy controls (C) and patients with OA or RA are shown in Figure 1.

#### 2.1.3. Endocannabinoid Analysis

**Figure 2:**
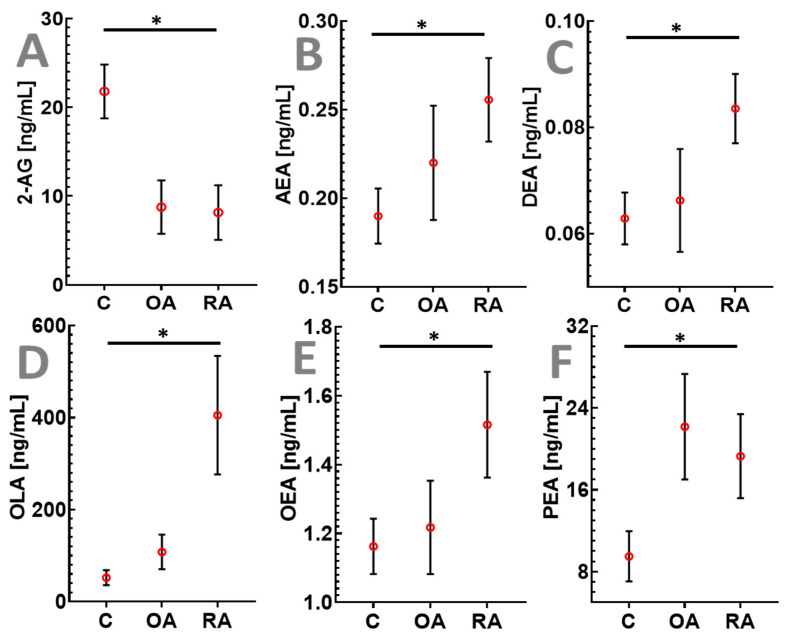
Changes in endocannabinoids and congeners that are broken down by monoacylglycerol lipase (2-AG) and fatty-acid amide hydrolase (AEA, DEA, OLA, OEA, and PEA) in osteoarthritis (OA) and rheumatoid arthritis (RA) compared to healthy controls (C). 2-AG (top left, (**A**)), AEA, (top center, (**B**)), DEA (top right, (**C**)), OLA (bottom left, (**D**)), OEA (bottom center, (**E**)), and PEA (bottom right, (**F**)) plasma levels in osteoarthritis (n = 17), rheumatoid arthritis (n = 25) and in age-matched healthy controls (n = 37). Data are shown as mean ± SEM. * FDR < 0.05. Significance for the comparison of OA versus healthy controls is not displayed since these were statistically non-significant (FDR > 0.05).

**Figure 3:**
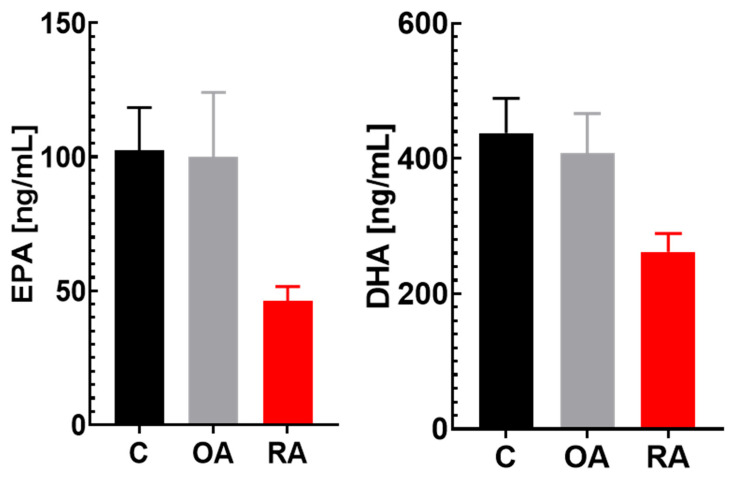
Plasma levels of fatty acids EPA (eicosapentaenoic acid) and DHA (docosahexaenoic acid) that make up the Omega-3 Index (O3I), a cardiovascular risk marker. Study groups: healthy controls (C, n = 37), patients with osteoarthritis (OA, n = 17), and patients with rheumatoid arthritis (RA, n = 25). Data are shown as mean ± SEM.

Plasma samples were analyzed using a slight modification of a previously published assay [42] and included 16 endocannabinoids. ANOVA analysis followed by an FDR test revealed that four endocannabinoids, namely, 2-arachidonoylglycol (2-AG), anandamide (AEA), oleamide (OLA), and docosatetraenoylethanolamide (DEA), and plasma levels were statistically significantly (FDR < 0.05) different between the study groups (Figure 2 and Figure 3). The N-acylethanolamines (NAEs) oleoylethanolamide (OEA) and palmitoylethanolamide (PEA) also showed similar trends (LSD *p* < 0.05; FDR > 0.05) to AEA and OLA (Figure 3). 2-AG levels (Figure 2) were lower in OA (8.8 ± 3.0 ng/mL; FDR = 0.066) and statistically significantly lower in RA patients (8.2 ± 3.1 ng/mL; FDR = 0.015) as compared to the healthy control group (21.7 ± 3.0 ng/mL). In contrast to 2-AG, endocannabinoids that belong to the group of N-acylethanolamines including AEA (FDR = 0.036), OEA (FDR = 0.050), DEA (FDR = 0.035), and PEA (FDR = 0.051) were increased in RA versus healthy subjects (Figure 2). Plasma concentrations of oleamide, which is also an FAAH substrate similar to the N-acylethanolamines mentioned above, were also significantly higher in the RA cohort (405 ± 128; FDR = 0.015) as compared to healthy participants (52 ± 16).

#### 2.1.4. Bioactive Lipids/Oxylipins

The analysis of bioactive lipids quantified 129 oxylipins including prostaglandins, leukotrienes, oxidated polyunsaturated fatty acids, resolvins, lipoxins, and other bioactive lipid mediators. Fifty-two (52) of these bioactive lipids were in the quantifiable range for the plasma samples analyzed during this study. 11-hydroxydocosahexaenoic acid (11-HDoHE; FDR = 0.038) was statistically significantly increased in RA compared to controls, and tetranor 12-hydroxyeicosatetraenoic acid (tetranor 12-HETE; FDR = 0.270), 5-iso prostaglandin F2α-VI (5-isoPGF2a VI; FDR = 0.069), and 8-hydroxyeicosatrienoic acid (8-HETrE; FDR = 0.312) showed trends (post hoc LSD *p* < 0.05; FDR > 0.05) of increased plasma concentrations in patients with RA as compared to age-matched healthy controls (see Supplementary Figure S1). These analytes also showed trends of higher concentrations in patients with OA; however, these are less pronounced and without reaching statistical significance. Furthermore, in patients with RA, significantly lower plasma concentrations of 9-oxo-octadecadienoic acid (9oxoODE; FDR = 0.042) were observed. There was a trend of decreased plasma levels observed for 20-hydroxyeicosatetraenoic acid (20-HETE; FDR = 0.069) and 14(15)-epoxyeicosatrienoic acid (14,15-EET; FDR = 0.259), of which the latter was statistically significantly lower in patients with OA (FDR = 0.039) as well (please see Supplementary Figure S1). Plasma levels of the ω-3 fatty acids EPA (eicosapentaenoic acid) and DHA (docosahexaenoic acid), that are also used to calculate the Omega-3 Index (O3I) [43], an established biomarker for coronary heart diseases [43] and other conditions [44], were lower in patients with RA (−40% and −55%; FDR = 0.069 and FDR = 0.088, for DHA and EPA, respectively) as compared to healthy participants (Figure 3). Both fatty acids remained unaffected in patients with OA.

## 3. Discussion

Rheumatoid arthritis is a chronic autoimmune disease primarily affecting the joints and extra-articular tissue [37]. The pathogenesis of RA involves the infiltration of leukocytes into synovial tissue and an increase in the production of inflammatory cytokines, such as interleukin (IL)-6 and tumor necrosis factor (TNF), resulting in chronic synovial inflammation [37,45,46]. In the past, RA used to be associated with high rates of disability, morbidity, and mortality. Today, treatment has been revolutionized with the advent of conventional synthetic DMARDs [37]. Numerous randomized controlled trials have reported significant pain reduction associated with treatment with DMARDs, but many patients still experience clinically meaningful levels of remaining pain despite treatment [47,48,49]. In contrast, OA is often referred to as a joint disease with damage and loss of cartilage but is also a much more diverse disease with complex pathogenesis that affects all tissues within the joint [10,50]. OA also has a significant inflammatory component, and inflammation in OA is found in the synovial membrane and infrapatellar fat pad [51]. The endocannabinoid system (ECS) has been identified as a key regulator in pain and a potential therapeutic target to treat chronic inflammatory pain [25,52]. The effects of the ECS on pain in OA have been investigated in preclinical studies [53,54,55], but data in humans are less frequent and focus mostly on synovium. One study suggested a crosstalk between the ECS and eicosanoid producing enzymes (cyclooxygenases and lipoxygenases) in synovium [56]. It has been shown that CB_2_ receptors play an important role in arthritis in animal models. β-caryophyllene (BCP), a CB_2_ agonist, ameliorated arthritis through a crosstalk between CB_2_ and PPAR-γ in a collagen antibody-induced arthritis model in mice [57]. Although the ECS has been extensively investigated in preclinical models of RA and in patients with de novo RA, the potential differences in the ECS in patients with RA undergoing DMARD therapy have not yet been studied. In the present study, we investigated the ECS (endocannabinoid levels) and interconnected lipid mediator pathways (lipid mediator levels) in patients with RA or OA compared to age-matched healthy participants.

Increased pro-inflammatory cytokine levels in patients with de novo RA have been well established, but there is limited data available comparing cytokine levels in patients with RA undergoing DMARD therapy to healthy individuals. IL-6 and TNF-α may play crucial roles in the activity and severity of RA [39,40,41]. In the present study, there was a trend toward higher TNF-α levels in the RA cohort compared to healthy participants. However, this trend was not statistically significant (Figure 1). The IL-6 plasma levels were statistically significantly higher in the RA group compared to both the OA and the healthy control groups (Figure 1 and Supplementary Table S1). This indicated that even with DMARD treatment, key inflammatory mediators in RA still remain elevated. Nevertheless, as the present study did not have a longitudinal design, it remains unclear what the initial values for IL-6 and TNF-α were prior to treatment in this study group. DMARDs significantly reduce inflammation and circulating cytokine levels [58]. For example, methotrexate treatment resulted in the upregulation of IL-1ra and the down-regulation of IL-1β as compared to treatment non-responders [59]. This might explain why IL-1ra plasma concentrations were significantly higher and IL-1β plasma concentrations were lower in the RA cohort than in healthy participants. In addition to the effects of DMARDs on cytokine levels, other drugs mostly taken by study participants with RA and OA could also have had effects on cytokine concentrations such as the use of nonsteroidal anti-inflammatory drugs (NSAIDs) [60]. Statins (used by about half the patients in the OA cohort) as well as corticosteroids (used by about a quarter of the patients in the RA cohort) have also been shown to reduce cytokine production [61,62]. In addition, commonly used psychiatric drugs (including anti-depressants, anticonvulsants, and anti-anxiety drugs that were taken by several of the patients in the OA cohort) have been shown to reduce key inflammatory cytokines such as IL-6 and TNF-α [63].

The endocannabinoid analysis revealed lower levels of 2-AG in patients with RA and OA than in healthy participants (Figure 2). The levels of 2-AG are regulated by degradation via monoacylglycerol lipase (MAGL) [64] activity and by synthesis via diacylglycerol lipase (DAGL) [65]. Previous studies have shown increased levels of 2-AG in synovial fluid from patients with RA and OA undergoing total knee arthroplasty (TKA) [30]. In contrast to these findings, the results of the present study showed lower levels of 2-AG in plasma. Limited information is available regarding the drugs used by participants in the previously mentioned study [30]. 2-AG has been shown to have anti-inflammatory and antinociceptive/analgesic properties [66,67,68]. Persistent pain in patients with RA and OA undergoing therapy is recognized as a significant problem [69,70]. The low 2-AG plasma levels observed in the present study may contribute to persistent pain. Hence, the endocannabinoid system and the inhibition of MAGL leading to 2-AG increases might be a potential therapeutic target for the treatment of inflammation and residual pain in RA and OA. In contrast to 2-AG, higher levels of N-acylethanolamines (NAEs) including AEA, OLA, OEA, DEA, and PEA were observed in patients with RA or OA than in healthy participants (Figure 2). This group of endocannabinoids and congeners is mainly regulated by fatty acid amide hydrolase (FAAH) and N-acyl phosphatidylethanolamine phospholipase D (NAPE-PLD) [71,72]. The major endocannabinoid anandamide (AEA), which was found at higher concentrations in the plasma of patients with RA than in the plasma of healthy participants, acts centrally as a cannabinoid 1 receptor (CB_1_) agonist leading to analgesic effects and is also a partial CB_2_ agonist. There are studies showing that the activation of CB_1_ and CB_2_, which are expressed in human, mouse, and horse synoviocytes [30,73,74,75], can induce potent anti-inflammatory effects and modulate arthritic disease [73]. It has been shown in a collagen-induced arthritis model that prolonged CB_2_ receptor activation diminished collagen-induced arthritis severity, whereas acute CB_1_ receptor activation reduced hyperalgesia in the same model [3,76]. However, AEA also has vanilloid activity and is a TRPV1 receptor agonist in the periphery [72], leading to noxious stimuli/pain [71,72]. TRPV1 plays a central role in the pathology of RA. The excessive activation of TRPV1 leads to immune cell dysfunction and the excessive release of inflammatory factors that mediate inflammation in the body [77]. The activation of the TRPV1 channel mediated by neuropeptide release and microglial activation induces nociplastic pain after inflammation control. TRPV1 also plays an important role in angiogenesis and cartilage destruction [77]. On the other hand, it has been shown that FAAH inhibition can cause continuous TRPV1 activation, which leads to desensitization and might have analgesic and anti-inflammatory effects [78]. Thus, it is not possible to decide as to whether the net effect of the observed higher AEA plasma concentrations is pro- or antinociceptive, and further research into the role of AEA in RA is required.

Enzymatically oxidized lipids (oxylipins) are a specific group of biomolecules that function as key signaling mediators and hormones, regulating various cellular and physiological processes from metabolism and cell death to inflammation and the immune response [79]. The ECS is directly or indirectly interconnected to many of these pathways, and changes in the endocannabinoid tone result in changes in enzyme activities in the lipid mediator pathways, and vice versa [80,81]. In the present study, a specific and quantitative LC-MS/MS-based multi-analyte assay was used to investigate differences in bioactive oxylipins (Figure 3 and Supplementary Figure S1). A group of oxylipins including 9-oxoODE and 14,15-EET were lower in the plasma of patients with RA or OA as compared to healthy participants. There is limited information on 9-oxoODE and 14,15-EET in the literature in the context of arthritis. One recent study investigating the benefits of a isocaloric anti-inflammatory diet (foods high in omega-3 fatty acids) on RA reported differences in 9-oxoODE in participants that responded to the diet, indicating that 9-oxoODE levels might be associated with omega-3 fatty acid concentrations [82]. These omega-3 fatty acids were very low in RA patients in our study (see below). Fischer et al. demonstrated that in healthy volunteers treated with EPA/DHA supplements, with an increase in the Omega-3 Index from about four to eight times, there was an increase in EPA-derived CYP-dependent epoxy metabolites (including 14,15-EET) [83,84]. Interestingly, 9-oxoODE and 14,15-EET have been described in the literature as possessing pro-resolving, anti-inflammatory, and vasodilative properties [85,86]. In summary, the decreased levels in these compounds might be contributors to inflammation and hypertension, which is common in RA [87,88]. 20-HETE, the plasma concentrations of which were lower in RA only, promotes endothelial activation and inflammation that involves the secretion of inflammatory cytokines (IL-6, IL-8, and TNFα) and the expression of adhesion molecules (ICAM/VCAM) [89]. Both 14,15-EET and 20-HETE are mostly formed by cytochrome P450 enzymes [83]. Higher levels of the two pro-inflammatory 12-lipoxygenase (12-LOX) products 11-HDoHE and tetranor-12-HETE (the main 12-HETE metabolite), as well as the pro-inflammatory 5-lipoxygenase (5-LOX) product 8-HETrE, were observed in patients with RA or OA than in healthy participants (Supplementary Figure S1). The changes in these pro-inflammatory oxylipins in RA have not been described before, and the underlying mechanism will require further investigation. There is evidence that 5-LOX, 15-LOX, and, to a lesser extent, 12-LOX are promising pharmacological targets for the therapy of OA and RA due to their involvement in the inflammatory process leading to degenerative joint diseases [90,91]. In fact, 5-LOX has been shown to drive the pyroptosis of CD4+ T cells and tissue inflammation in rheumatoid arthritis [92]. The fact that several 5-LOX and 12-LOX products are increased in OA and RA, albeit the participants underwent treatment, indicates that treatments targeting lipoxygenases (e.g., zileuton [92]) might be beneficial companion therapies to the current standard-of-care.

Omega-3 fatty acids have anti-inflammatory actions [93]. Clinical studies have shown that omega-3 fatty acids may have a modulatory effect on disease activity, namely, on the number of swollen and tender joints [93]. The omega-3 fatty acids EPA and DHA (Figure 3) are commonly used to calculate the Omega-3 Index (O3I) [43]. Both polyunsaturated fatty acids were significantly lower in patients with RA compared to heathy controls, indicating a decreased O3I in these patients. There was no difference between patients with OA and healthy participants. This finding confirms findings from a previous study in Korean women, which compared 100 female patients with RA with age-matched female participants [94]. Many preclinical and clinical studies have suggested omega-3 fatty acid supplementation to be beneficial for RA and to reduce inflammation and pain [93,95,96,97]. The results of the present study showed that there is still an omega-3 fatty acid deficiency in patients with RA undergoing treatment that might contribute to disease severity. This finding supports the notion that omega-3 supplementation might be beneficial to increase plasma omega-3 fatty acid levels in patients with RA, potentially resulting in improved inflammation and pain symptoms, as the literature suggests [97]. As mentioned before, several of the changes in oxylipins, including 14,15-EET, 9-oxoODE, and potentially LOX pathways [98], might be related to/resulting from the decreased Omega-3 Index observed in RA.

### Limitations

This was not a study investigating differences in endocannabinoids and bioactive oxylipins in de novo patients with RA and OA. The results of the present study provide the rationale for larger studies. These studies should also include de novo patients with RA and OA to allow conclusions about the pathophysiological role of ECS and oxylipins in RA and OA, independent of drug effects. Studies that include OA and RA subjects undergoing therapy should be stratified by drug regimens, which will require much larger study cohorts. Using the current study design, it is difficult to differentiate between the effects of medications and disease conditions, since treatments with DMARDs, NSAIDs, and other drugs were much more prevalent in the RA and OA study groups as compared to the age-matched healthy participants.

## 4. Materials and Methods

### 4.1. Patient/Volunteer Enrollment and Blood Draw

The study protocol and all its amendments were approved by the Colorado Multi-Institutional Review Board (COMIRB, Aurora, CO, USA, study #21-3357). Participants provided written informed consent, and the study was carried out in compliance with all applicable local and national rules, regulations, and policies, as well as in accordance with the Declaration of Helsinki and its amendments. Participants consisted of 25 patients with RA, 17 patients with OA, and 37 age- and gender-matched healthy participants. All participants completed a blood draw and provided information about their past 30-day medication use and other substance use patterns. Table 1 summarizes the study demographics. Inclusion criteria for the study included age between 30 and 70 years old, any gender or ethnicity, documented knee OA (according to the criteria of the American College of Rheumatology [99]) for the OA group, or a diagnosis of RA (fulfillment of the 1987 [100] or 2010 American College of Rheumatology/EULAR RA Classification criteria) for the RA group. Exclusion criteria included (A) a change in OA- or RA-related medications within the last 4 weeks, (B) the use of cannabis or hemp products within the last 2 weeks, (C) instable co-morbid conditions including associated changes in medications within the prior 2 weeks, and (D) a body mass index (BMI) of over 42. Fasting prior to blood draw was not a requirement. Blood samples for analysis were collected via venous blood draw using K_2_EDTA (ethylenediaminetetraacetic acid) as anti-coagulant. Plasma was generated using a standard centrifugation procedure. One aliquot of plasma samples was acidified for the preservation of endocannabinoids, as previously described by our group [42].

### 4.2. Cytokine Multiplex Analysis

A commercially available magnetic bead-based Luminex MAGPIX assay (Luminex Performance Assay multiplex kits, Catalog Number LKTM014B, R&D Systems, Minneapolis, MN, USA) was performed following the manufacturer’s instructions. Each plasma sample was analyzed for 46 cytokines using the Human XL Cytokine Luminex Performance Assay 46-plex Fixed Panel, including interleukins, interferons, growth factors, chemokines, and others. Analysis was performed in duplicate (singlet analysis on one plate, repeated on two different days).

### 4.3. Endocannabinoid Analysis

Endocannabinoid analysis was performed using a validated LC-MS/MS assay previously described by our group [42,101]. Briefly, aliquots of 200 μL of the calibrator, quality control, or study sample were transferred into a 1.5 mL polypropylene vial with conical bottom and snap-on lid. Eight hundred microliters of acetonitrile containing deuterated internal standards were added, and the samples were mixed for 10 min on a vortex mixer (Multitube vortexer, Fisher Scientific, Waltham, MA, USA). The samples were centrifuged at 25,000× *g* and 4 °C for 10 min (MR 23i centrifuge with a fiberlite rotor, Thermo Scientific, Waltham, MA, USA). Seven hundred fifty microliters of the supernatant were transferred into HPLC vials. Then, 450 μL of 0.1% formic acid in water was added, and the extracts were briefly vortexed. The sample extracts were placed into the HPLC autosampler for subsequent analysis. After the extraction of the plasma samples, endocannabinoids were quantified using a Sciex API 5500+ mass spectrometer (Sciex, Marlborough, MA, USA) equipped with an Agilent 1260 HPLC system (Agilent Technologies, Santa Clara, CA, USA). Sixteen (16) endocannabinoids were quantified including N-acylethanolamines (e.g., anandamide), 2-acylglycerols (e.g., 2-AG), N-acyl dopamines (e.g., N-arachidonoyl dopamine and NADA), and primary fatty acid amines (e.g., oleamide). For further details regarding the chromatographic process, instrument settings, quantification of endocannabinoids, assay performance parameters, and validation results, please see Sempio et al. [42].

### 4.4. Bioactive Lipid/Oxylipin Analysis

Bioactive lipid mediators were analyzed in plasma samples using a multi-analyte LC-MS/MS assay. The assay analyzed 129 lipid mediators including prostaglandins, leukotrienes, resolvins, and oxylipins and was a modification of previously described methods [102,103,104,105]. Briefly, 100 µL plasma was aliquoted into 1.5 mL micro centrifuge tubes. Ten (10) µL of a mix of isotope-labeled internal standards was added to each sample and vortexed briefly. Then, 300 µL methanol/acetonitrile 1/1 was added for protein precipitation. The samples were vortexed for 10 min and then centrifuged for 10 min at 25,000× *g*. In total, 250 µL of supernatant was used and diluted with 416 µL water and directly injected into an inline extraction-enabled HPLC–tandem mass spectrometry system. The extracts were analyzed using an Agilent 1290 series HPLC (Agilent Technologies, Santa Clara, CA, USA) interfaced with an Sciex API7500 mass spectrometer system via an OptiFlow Pro Source operated in the negative electrospray ionization mode (Sciex, Marlborough, MA, USA). This approach has been proven to deliver reliable, quantitative, and ultrasensitive results for the analysis of this group of small-molecule lipid mediators [103,104,105,106,107].

### 4.5. Statistical Analysis

Results are expressed as means ± standard error of the mean (SEM) of n observations, where n represents the number of participants. Sample sizes were not pre-determined by statistical methods. The normality of distribution was assessed before hypothesis testing where deemed necessary. A two-way ANOVA in combination with an LSD post hoc test was carried out to test if endocannabinoid, lipid mediator, or cytokine levels differed amongst the three study groups (healthy controls, OA, and RA). The Benjamini–Hochberg procedure was used to control the false discovery rate (FDR) [108,109,110]. The level of significance was set to an alpha of 0.05. Analyses of data were conducted using IBM SPSS software (version 29, IBM, Armonk, NY, USA). To determine the FDR, Metaboanalyst software (version 6.0, www.metaboanalyst.ca, accessed on 2 June 2025) was used [111].

## 5. Conclusions

Overall, our results suggest the modulation of endocannabinoids in inflammation and pain in patients with RA and OA and may present a potential treatment target. Plasma concentrations of 2-AG, which is a key regulator of nociception and inflammation, were lower in patients with RA or OA, and it seems reasonable to expect that this is a mechanistic contributor to residual inflammation in patients with RA and OA receiving treatment. The plasma concentrations of other endocannabinoids, including anandamide and other NAEs, were higher in patients with RA and OA receiving treatment than in healthy participants and might be an indicator of decreased FAAH (fatty acid amide hydrolase) activity in RA and, to a lesser degree, in OA. The plasma concentrations of several pro-inflammatory lipoxygenase-derived oxylipins were higher in patients with OA than in healthy participants and, to a greater extent, in patients with RA. These findings have not been described in clinical studies before, and further investigation into the mechanisms is required. Decreased levels of the main omega-3 fatty acids EPA and DHA were observed in patients with RA but not with OA, and indicate a deficiency in these important nutritional anti-inflammatory, polyunsaturated fatty acids.

## Figures and Tables

**Table 1:** Study demographics.

		Overall (n = 80)	Healthy Participants (n = 37)	Osteoarthritis (n = 18)	Rheumatoid Arthritis (n = 25)
Race	W/B/A/O	54/5/6/15 (68%/6%/8%/19%)	26/1/4/6 (70%/3%/11%/16%)	12/3/0/3 (67%/18%/0%/18%)	16/1/2/6 (64%/4%/8%/24%)
Ethnicity	non-Hispanic/Hispanic/unknown	64/7/9 (80%/9%/11%)	29/1/7 (78%/3%/19%)	17/0/1 (94%/0%/6%)	18/6/1 (72%/24%/4%)
Sex	female/male	53/27 (66%/34%)	22/15 (59%/41%)	15/3 (83%/17%)	16/9 (64%/36%)
Age	[years]	56 ± 11	57 ± 8	62 ± 7	52 ± 12
BMI	[kg/m^2^]	28 ± 9	30 ± 14	30 ± 7	25 ± 4

Abbreviations: W, White or Caucasian; B, Black or African American; A, Asian; O, Other.

**Table 2:** Medications used by study participants.

	Healthy Participants (n = 37)	Osteoarthritis (n = 18)	Rheumatoid Arthritis (n = 25)
DMARDs	0	1	25
NSAIDs	1	9	14
Corticosteroids	0	0	6
Antihistamines	3	0	1
Statins and cholesterol-lowering medications	0	8	8
Other pain medications including opioids	0	2	3
Antidepressants, anti-anxiety, and anticonvulsant	3	6	9
Diabetes medications	0	4	4
Hypertension medications	3	8	11
Hypothyroidism medications	6	1	7
Other medications *	2	6	10

Abbreviations: DMARDs, disease-modifying antirheumatic drugs; NSAIDs, nonsteroidal anti-inflammatory drugs. * Other medications include, but are not limited to, antivirals, antacids, antipsychotic, antiepileptic, migraine, sleep, and diarrhea medications.

## Data Availability

The data presented in this study are available on request from the corresponding author.

## Supplementary Materials

The supporting information can be downloaded at: https://www.mdpi.com/article/10.3390/ijms26125707/s1.
