# Supplementary material for: Endocannabinoid Tone and Oxylipins in Rheumatoid Arthritis and Osteoarthritis—A Novel Target for the Treatment of Pain and Inflammation?
Source: Int J Mol Sci. 2025 Jun 14;26(12):5707. doi: 10.3390/ijms26125707 (PMC12192655; doi:10.3390/ijms26125707)
Supplement: Supplementary file 1 [file ijms-26-05707-s001.zip › ijms-3636205-supplementary.pdf]

Supplementary Materials

Table S1. Cytokine plasma concentrations in patients with rheumatoid arthritis (RA), patients with osteoarthritis (OA), and healthy volunteers (C).

Cytokine	LLOQ	C (n = 37)	OA (n = 18)	OA vs. C	RA (n = 25)	RA vs. C
	[pg/mL]	[pg/mL]	[pg/mL]	FDR	[pg/mL]	FDR
**EGF**	7.04	26.4 ± 0.1	13.5 ± 0.2	0.212	9.3 ± 0.1 *	0.020
**Eotaxin**	23.7	102.2 ± 0.1	102.2 ± 0.6	0.998	115.6 ± 0.4	0.266
**Flt-3 Ligand**	25.4	59.3 ± 0.1	62.2 ± 0.4	0.926	81 ± 0.2 *	0.020
**GROβ**	11.0	585.3 ± 2.1	300.6 ± 4.1	0.097	270.2 ± 3.9 *	0.032
**IFN-γ**	0.49	1.21 ± 0.01	0.95 ± 0.01	0.646	2.27 ± 0.04	0.285
**IL-1α †**	10.3	4.93 ± 0.02	3.27 ± 0.03	0.392	3.81 ± 0.03	0.345
**IL-1β †**	3.42	2.35 ± 0.01	0.99 ± 0.02	0.241	1.15 ± 0.01	0.285
**IL-1ra**	11.0	294.2 ± 0.7	351.5 ± 3.1 *	0.047	543.7 ± 3.9 *	0.034
**IL-6**	8.4	7.38 ± 0.02	6.31 ± 0.05	0.536	13.8 ± 0.14 *	0.020
**IL-13**	30.8	57.2 ± 0.1	51.7 ± 0.2	0.536	53.7 ± 0.1	0.059
**IP-10**	2.73	82.5 ± 0.3	83.1 ± 0.7	0.998	101.9 ± 0.5	0.285
**MCP-1**	5.23	82.3 ± 0.1	94.7 ± 0.4	0.270	100.1 ± 0.2 *	0.020
**MIP-1α**	5.23	28 ± 0	26.8 ± 0.1	0.926	24.7 ± 0.1	0.285
**MIP-3α**	2.22	12.3 ± 0	10.2 ± 0.1	0.543	84.6 ± 2.6	0.285
**MIP-3β**	4.49	90.3 ± 0.5	88 ± 1.1	0.998	102.8 ± 0.3	0.575
**PDGF-AA**	8.92	937.1 ± 3.5	339.9 ± 4.8	0.044	350.7 ± 4.1 *	0.020
**PDGF-AB/BB**	8.44	473.3 ± 1.7	203.6 ± 3.3	0.051	212.1 ± 3.2 *	0.032
**PD-L1/B7-H1**	6.58	41.4 ± 0.1	41.3 ± 0.2	0.998	54.5 ± 0.2 *	0.032
**RANTES**	315.7	57,704 ± 179	25,712 ± 399 *	0.044	25,805 ± 371 *	0.020
**TNF-α**	11.40	7.12 ± 0.01	7.33 ± 0.06	0.998	10.04 ± 0.07	0.110
**TNF-β**	0.86	4.16 ± 0.01	4.86 ± 0.05	0.646	5.11 ± 0.04	0.345
**VEGF**	6.43	68.5 ± 0.2	48.2 ± 0.3	0.270	57.3 ± 0.2	0.345

Abbreviations: LC: lowest calibrator; EGF, epidermal growth factor; Flt3, FMS-like tyrosine kinase 3; GRO, growth-regulated protein; IFN, interferon; IL, interleukin; IP, interferon gamma-induced protein; MCP, monocyte chemoattractant protein; MIP, macrophage inflammatory protein; PGF, platelet-derived growth factor; PD-L1, programmed death ligand-1; RANTES, regulated on activation, normal T-cell expressed and secreted; TNF, tumor necrosis factor; VEGF, vascular endothelial growth factor. Data are shown as mean ± SEM. * FDR < 0.05. The symbol † indicates that values are below the lowest calibrator, but above the limit of detection.

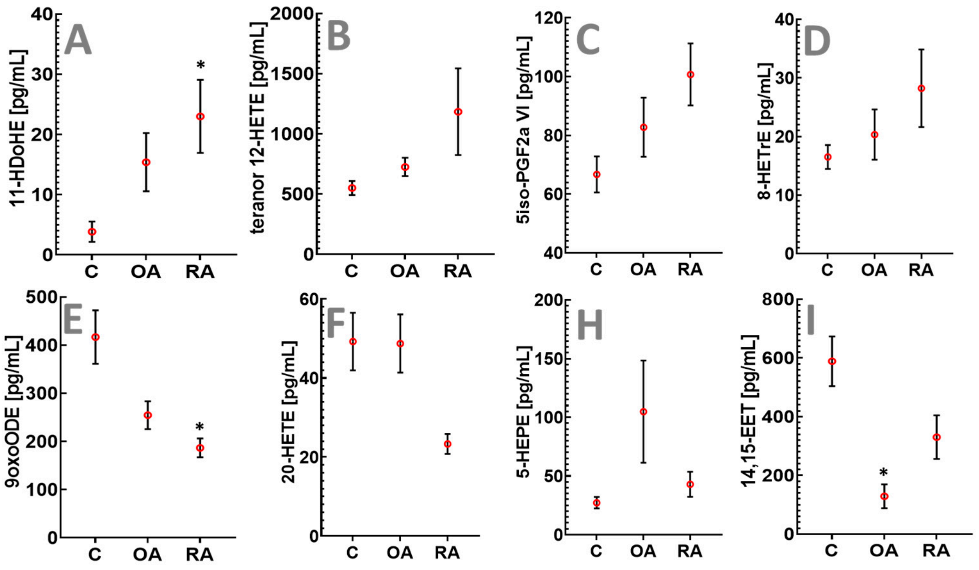

Figure S1. Key lipid mediators in OA and RA. Comparison of plasma concentration of key lipid mediators in OA (n = 17) and RA (n = 25) compared to age-matched healthy controls (C, n = 37). Data are shown as mean ± SEM. * FDR < 0.05.
